# Quantifying the cooperative subunit action in a multimeric membrane receptor

**DOI:** 10.1038/srep20974

**Published:** 2016-02-09

**Authors:** Nisa Wongsamitkul, Vasilica Nache, Thomas Eick, Sabine Hummert, Eckhard Schulz, Ralf Schmauder, Jana Schirmeyer, Thomas Zimmer, Klaus Benndorf

**Affiliations:** 1 Institut für Physiologie II, Universitätsklinikum Jena, Friedrich-Schiller-Universität Jena, 07743 Jena, Germany; 2 Hochschule Schmalkalden, Fakultät Elektrotechnik, Blechhammer, 98574 Schmalkalden, Germany

## Abstract

In multimeric membrane receptors the cooperative action of the subunits prevents exact knowledge about the operation and the interaction of the individual subunits. We propose a method that permits quantification of ligand binding to and activation effects of the individual binding sites in a multimeric membrane receptor. The power of this method is demonstrated by gaining detailed insight into the subunit action in olfactory cyclic nucleotide-gated CNGA2 ion channels.

A large number of membrane receptors are multimeric proteins composed of a well-defined number of equal or different subunits. When one or more ligands bind to a multimeric receptor, it exerts a conformational change, resulting either in modulating a conductance for ions or a changed readiness to interact with other proteins. Up to now for no multimeric receptor the molecular events upon activation have been fully elucidated. The main reason for this lack of exact knowledge is that receptor function is governed by subunits cooperating with each other in a complex manner^1^.

For methodical approaches, ligand-gated ion channels provide the unique feature that the final conformational change, the pore opening, can be directly excellently quantified by the patch-clamp technique^2^, often by using single-channel analysis and hidden Markovian state models^3^.

CNG channels are tetrameric membrane receptors that generate a non-selective conductance for cations^4^,^5^. The channels are activated by the cytosolic cyclic nucleotides cAMP or cGMP, binding to specific cyclic nucleotide binding domains (CNBDs) in each subunit^6^. Wild-type olfactory CNG channels are composed of three types of subunits^7^ out of which only the CNGA2 subunits can form functional homotetrameric channels on their own when expressed in heterologous systems^8^,^9^. Homotetrameric CNGA2 channels are very useful model systems for studying elementary biophysical processes^10^,^11^, though also for these channels the subunit action upon channel activation is still a mysteriously complex and cooperative process, resulting in respectively branched Markovian models^10^,^12^. Other experimental findings further substantiate the complexity of the activation process, including the facts that Hill coefficients are two^13^ or even higher^14^ and that the pore opening and closure is a one-step process though four subunits are involved. To shed further light into the activation process, it is therefore still highly desirable to develop new methods monitoring the operation of the individual subunits in a functional channel.

Here we present a powerful and straightforward approach to analyze both the binding affinity and the gating effect of the individual subunits in a functional multimeric ionotropic receptor channel. CNGA2 channels were functionally expressed in *Xenopus* oocytes and currents induced by the cGMP binding were measured in inside-out patches. We also used subunits with a disabled binding site by inferring the mutation R538E (*mut*)^15^ which shifted the apparent affinity in the concentration-activation relationship of homotetrameric *mut*-channels (4×*mut*) by a factor of 315 to higher concentrations with respect to homotetrameric *wt*-channels (4×*wt*; Fig. 1a) leaving the Hill coefficient unaffected (Supplementary Table 1a). We next constructed tetrameric concatamers with either four *wt*- or four *mut*-subunits and tested whether these *wt*-*wt*-*wt*-*wt* and *mut*-*mut*-*mut*-*mut* channels are activated closely similar to 4×*wt* and 4×*mut* channels, respectively. This was substantiated by the following results: (1) The respective concentration-activation relationships match each other (Fig. 1a). (2) Co-expressing *wt-wt-wt-wt* with *mut-mut-mut-mut* channels (cRNA ratio 1:1) produced a concentration-activation relationship containing two well determined components with characteristics specific for each channel entity, suggesting that the concatameric channels operate independently (Fig. 1b, Supplementary Table 1b). (3) At respectively saturating cGMP, the amplitude of the single-channel current in both *wt*-*wt*-*wt*-*wt* and *mut*-*mut*-*mut*-*mut* channels was indistinguishable from that in 4×*wt*-channels (Supplementary Fig. 1a). (4) The open probability at saturating cGMP in *wt*-*wt*-*wt*-*wt* (0.99 ± 0.002; 100 μM cGMP; *N* = 4) and *mut*-*mut*-*mut*-*mut* channels (0.99 ± 0.003; 5 mM cGMP; *N* = 5) was similarly close to unity as in 4×*wt* channels (0.99 ± 0.002; 100 μM cGMP; *N* = 4).

We then constructed concatamers with increasing numbers of mutated subunits in the background of wild-type subunits, i.e*. mut-wt-wt-wt, mut-mut-wt-wt, and mut-mut-mut-wt*. The concentration-response relationships for these channels were systematically shifted to higher cGMP concentrations in proportion to the number of incorporated *mut*-subunits (Fig. 1a). For concatamers with either one or two *wt*-subunits we also showed that, surprisingly, the channel function does not depend on the position of the respective subunits (Fig. 1c, Supplementary Table 1c). In the following analysis we included as representative concatamers *mut-mut-wt-wt* and *mut-mut-mut-wt*.

In the concentration-activation relationships of the respective channels the slope systematically decreased when decreasing the number of *wt*-subunits from four to one (Fig. 1a). Apart from *mut*-*mut*-*mut*-*wt* channels, all concentration-activation relationships could be described reasonably by a single Hill function (Equation 1) whereas *mut*-*mut*-*mut*-*wt* channels required the sum of two Hill functions (Equation 2), presumably mirroring the action of the only *wt*-subunit on the pore opening (high affinity component (*EC*_50,H_)) and that of the three *mut*-subunits (low affinity component (*EC*_50,L_), respectively.

To test whether or not the concentration-activation relationships considered so far represent exclusively effects of cGMP on the channel gating, and not on the unitary current, single-channel experiments were performed with *mut*-*mut*-*mut*-*wt* channels. The amplitude of the unitary currents (Supplementary Fig. 2) matched those of 4×*wt*, *wt*-*wt-wt-wt*, and *mut-mut-mut-mut* channels at saturating cGMP (Supplementary Fig. 1). Similar results were obtained with two *wt*-subunits in *mut*-*mut*-*wt*-*wt* (Supplementary Fig. 3). These results suggest that the channel pore operates as a whole and that an increasing number of occupied binding sites only increases the open probability. This conclusion is consistent with kinetic models containing only one concerted opening step of all subunits^10^,^16^.

Based on the above results, the concentration-activation relationships of the five properly operating concatameric channels (Fig. 1a) contain complex information about the intricate gating of the subunits. To adequately uncover this information we performed a global fit analysis by five Markovian submodels, one for each concatamer (4*wt*-submodel through 0*wt*-submodel; Fig. 2a). In each of these submodels four ligands can bind, quantified by the association constants *K*_Ax_ (x = 1…4), which are, according to the number of *wt*- and *mut*-subunits, subdivided in high-affinity constants, *K*_AxH_, and low-affinity constants, *K*_AxL_, respectively. At each degree of liganding the channel can open by a single step, quantified by an equilibrium constant for the closed-open isomerization *E*_x_ (x = 0…4). The five submodels are intimately coupled via their equilibrium constants. Due to microscopic reversibility^17^ the global fit contained only six free parameters (Supplementary Methods). Most favorably, an active effect of each subunit is available at two distant ligand concentrations, providing a considerable surplus of information.

The coupled submodels fully describe the series of concentration-activation relationships (Fig. 2b; for parameters see Table 1). *E*_0_ and *E*_4_ were determined separately in single-channel experiments at zero and saturating cGMP in *wt*-*wt*-*wt*-*wt* channels. Because the fit did not permit to distinguish *K*_A3H_ from *K*_A4H_, *K*_A3L_ from *K*_A4L_ and *E*_3_ from *E*_4_, we set these respective constants equal. With this restriction the equilibrium constants were determined with formidable accuracy. These results show to what extent *K*_AxH_ and *E*_x_ depend on the number of ligands. In line with a previous approach^10^, binding of the first ligand generates already noticeable channel opening (*E*_1_ = 3.59 × 10^−1^).

Knowing the equilibrium constants for all binding and open-closed isomerizations provides the possibility to evaluate for all concatamers the occupancy of the states as function of the cGMP concentration. Consider e.g. the occupancies in the 1*wt*-submodel (Fig. 2c). The probabilities to occupy a binding site are extended along the abscissa but do also considerably overlap. Moreover, the strong disabling effect of the mutation R538E in the *mut*-subunits causes that, apart from the empty states (C_0_, O_0_), only states with an occupied *wt*-subunit are relevantly populated. Another result is that up to ~3 μM cGMP only the *wt*-subunit is occupied, generating measurable channel activity.

In summary, we propose a powerful method to learn more about how individual subunits in a multimeric ion channel work in the presence of the other subunits. The asset of the method comes from the fact that the different number of four to zero *mut*-subunits does not only extend the concentration-activation relationships along the concentration axis but also characteristically shapes them, thereby providing valuable information.

To some extent this analysis is related to the well-established double-mutant cycle analysis^18^ that analyzes Gibbs free energy differences for two single and the corresponding double mutant to specify free energies of interaction^19^. In case of ion channels the *EC*_50_ values are regularly used to estimate these free energies of interaction^20^. Compared to such analyses, our approach is more comprehensive by including single, double, triple, and quadruple mutated channels as well as the specific effects of these mutations on the curvature of the concentration-activation relationships.

The proposed method bears potential to unravel also the properties of native heterotetrameric olfactory 2×CNGA2:CNGA4:CNGB1b channels, which would permit to address the question which of the subunits operates the channels at physiologically low cyclic-nucleotide concentrations. Besides CNG channels the method should be applicable also to any other channel. It seems to be also possible to adapt this method to analyze single-channel activity. And, beyond electrophysiological approaches, even metabotropic multimeric membrane receptors should be accessible for this approach if an appropriate read-out for the receptor activation is available, e.g. by inferring donor and acceptor fluorophores in the subunits and evaluating Förster resonance energy transfer (FRET)^21^.

## Methods

### Oocyte Preparation and cRNA Injection

Oocytes were obtained surgically under anesthesia (0.3% 3-aminobenzoic acid ethyl ester) from adult females of *Xenopus laevis*^11^. The procedures had approval from the authorized animal ethical committee of the Friedrich Schiller University Jena. The methods were carried out in accordance with the approved guidelines.

### Molecular biology

The rCNGA2 tetrameric concatamers were obtained by joining the coding sequences of rat CNGA2 subunits via the short linker sequence GSA into a single ORF. To facilitate the concatenation procedure, we first introduced by PCR a *Bcl*I site in front of the CNGA2 (*wt*) start codon in vector pGEMHEnew^22^, and an in-frame *BamH*I site in front of the respective stop codon. These sites were also introduced into the pGEMHEnew vector coding for the R538E mutant (*mut*). In a second step, we constructed various dimers by inserting a *Bcl*I/*EcoR*I fragment of *wt* or *mut* into the *BamH*I/*EcoR*I sites of the modified pGEMHEnew vector containing either the full-length *wt* or *mut* sequences. Finally, two dimers were coupled using the same cloning strategy. The sizes of the dimeric and tetrameric DNA were confirmed by restriction enzyme mapping and gel electrophoresis. The linkers and flanking sequences were sequenced to verify that there were no secondary mutations and that the R538E mutation was correctly inserted. The cRNAs were transcribed from cDNAs *in vitro* with the mMESSAGE mMACHINE T7 Kit (Ambion, Austin, TX).

### Electrophysiology

For obtaining steady-state concentration-activation relationships macroscopic currents were recorded from inside-out patches with a standard patch-clamp technique. The patch pipettes were pulled from quartz tubing (P-2000, Sutter Instrument, Novato, USA) with an outer and inner diameter of 1.0 and 0.7 mm (VITROCOM, New Jersey, USA). The pipette resistance was 0.5–1.7 MΩ. The bath and pipette solution contained (in mM): 150 KCl, 1 EGTA, 10 Hepes (pH 7.4 with KOH). Recordings were performed with an Axopatch 200A amplifier (Axon Instruments, Foster City, CA). Electrophysiology was controlled by the ISO2 hard- and software (MFK, Niedernhausen, Germany). The sampling rate was 5 kHz and the filter of the amplifier (4-pole Bessel) was set to 2 kHz. For single-channel measurements, the patch pipettes were pulled from quartz tubing with an outer and inner diameter of 1.0 and 0.5 mm (VITROCOM, New Jersey, USA). The pipette resistance was 5.0–12.0 MΩ. The sampling rate was 20 kHz and the filter of the amplifier (4-pole Bessel) was set to 5 kHz. The pipette solution contained (in mM): 150 KCl, 1 EGTA, 5 Hepes (pH 7.4 with KOH) and 200 μM niflumic acid to block endogenous chloride channels. The recording voltage was +100 mV.

### Data analysis

If not otherwise noted, concentration-activation relationships were fitted with OriginPro 8G (Northampton, USA) by


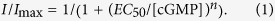


*I* is the actual current amplitude, and *I*_max_ the maximum current amplitude at saturating cGMP specified for each patch. The saturating cGMP concentrations ranged from 100 μM to 5 mM. *EC*_50_ is the cGMP concentration generating the half maximum current and *n* the Hill coefficient.

Some concentration-activation relationships required the sum of a high (H) and a low affinity (L) component:


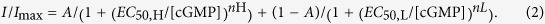


*A* is the fraction of the high affinity component.

Amplitude histograms of the single channel activity were fitted by respective sums of Gaussian functions. The global fit of the concentration-activation relationships is described in Supplementary Methods.

**Statistics.** Data are given as mean ± s.e.m.

## Additional Information

**How to cite this article**: Wongsamitkul, N. *et al.* Quantifying the cooperative subunit action in a multimeric membrane receptor. *Sci. Rep.* **6**, 20974; doi: 10.1038/srep20974 (2016).

## Supplementary Material

Supplementary Information

## Figures and Tables

**Figure 1:**
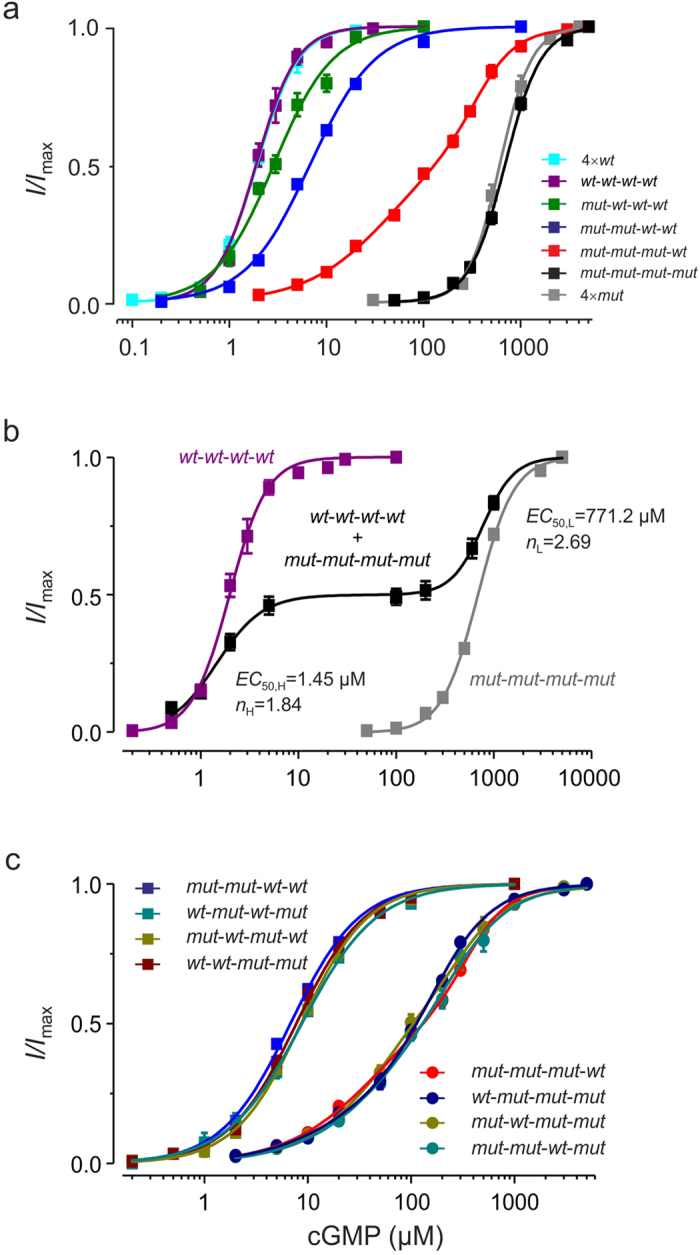
Concentration-activation relationships of CNGA2 concatamers. (**a**) Effect of an increasing number of *mut*-subunits. The channels are either formed by four monomers (4×*wt*, 4×*mut*) or by tetrameric concatamers (*wt*-*wt*-*wt*-*wt*, *mut*-*wt*-*wt*-*wt*, *mut*-*mut*-*wt*-*wt*, *mut*-*mut*-*mut*-*wt*, *mut*-*mut*-*mut*-*mut*). All fit parameters are provided by Supplementary Table 1a. (**b**) Concatamers assemble as tetrameric channels. Expression of either *wt*-*wt*-*wt*-*wt* or *mut-mut-mut-mut* channels alone or together with a cRNA ratio 1:1 (*N* = 14–21). All fit parameters are provided by Supplementary Table 1b. (**c**) The position of *wt*-subunits is irrelevant for the concatamer function. The concentration-activation relationships of four concatamers with two *wt*-subunits and four concatamers with one *wt*-subunit are plotted. The fit parameters are given in Supplementary Table 1c. The relationships for the concatamers with one *wt*-subunit were indistinguishable as were the relationships for the concatamers with two *wt*-subunits (multidimensional *t*-test with Holm correction, *p*-value = 1).

**Figure 2:**
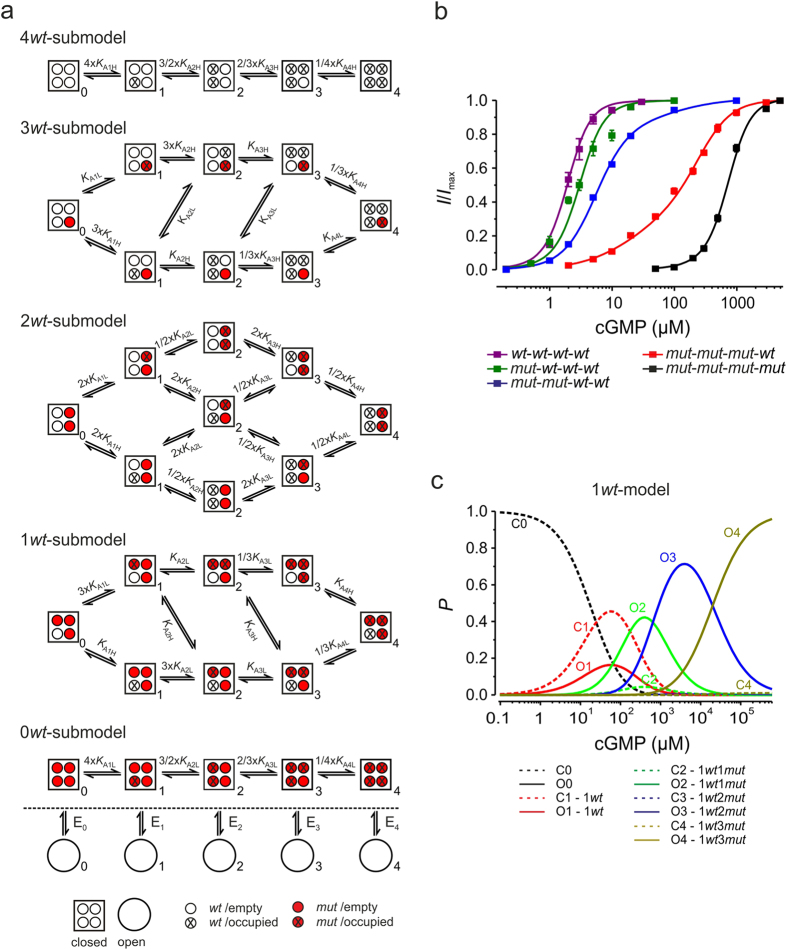
Global fit of concentration-activation relationships from five CNGA2 concatamers. (**a**) Markovian submodels describing the activation gating of five concatamers. The closed-open isomerizations, including their equilibrium constants, *E*_0_….*E*_4_, are indicated only once at the bottom. They are the same for each equally liganded state. *K*_A1H_, *K*_A2H_, *K*_A3H_, *K*_A4H_, *K*_A1L_, *K*_A2L_, *K*_A3L_, and *K*_A4L_ are the equilibrium association constants for the four high and low affinity binding sites, respectively. *E*_0_ and *E*_4_ were set to 1.7 × 10^−5^ and 9.9 × 10^1^ according to the single-channel experiments. (**b**) Global fit of the data points of the five concatamers shown in **a**. The values of the equilibrium constants are provided by Table 1. (**c**) Occupancy of the states (*P*) predicted by the 1*wt*-submodel as function of the [cGMP].

**Table 1:** Equilibrium constants determined by the global fit.

Equilibrium constant	Dimension	Mean	s.e.m.	CV%
*K*_A1H_	M^−1^	4.05 × 10^4^	0.15 × 10^4^	3.76
*K*_A2H_	M^−1^	7.90 × 10^4^	0.65 × 10^4^	8.26
*K*_A3H_ = *K*_A4H_	M^−1^	5.92 × 10^4^	0.49 × 10^4^	8.33
*K*_A1L_	M^−1^	1.03 × 10^2^	0.04 × 10^2^	4.18
*K*_A2L_	M^−1^	2.01 × 10^2^	0.16 × 10^2^	8.08
*K*_A3L_ = *K*_A4L_	M^−1^	1.51 × 10^2^	0.12 × 10^2^	8.06
*E*_0_	—	1.70 × 10^−5^	—	—
*E*_1_	—	3.59 × 10^−1^	0.14 × 10^−1^	3.97
*E*_2_	—	9.58 × 10^0^	0.61 × 10^0^	6.35
*E*_3_ = *E*_4_	—	9.90 × 10^1^	—	—

The coupled submodels shown in Figure 2a were globally fitted to the respective concentration-activation relationships (Fig. 2b). Parameters were the equilibrium association constants for the four high-affinity binding sites, *K*_A1H_, *K*_A2H_, *K*_A3H_, *K*_A4H_, the equilibrium association constants for the four low-affinity binding sites, *K*_A1L_, *K*_A2L_, *K*_A3L_, *K*_A4L_, and the three equilibrium constants for the closed-open isomerizations *E*_1_, *E*_2_, *E*_3_. *E*_0_ and *E*_4_ were determined by single-channel experiments to be 1.7 × 10^−5^ and *E*_4_ to 9.9 × 10^1^. To increase the constraints, we assumed *K*_A3H_ = *K*_A4H_, *K*_A3L_ = *K*_A4L_, and *E*_3_ = *E*_4_. The values are given as mean ± s.e.m. CV% indicates the error in %. *χ*^2^ was 123.91.
