# Corrigendum: Quantifying the cooperative subunit action in a multimeric membrane receptor

**DOI:** 10.1038/srep47001

**Published:** 2018-06-28

**Authors:** Nisa Wongsamitkul, Vasilica Nache, Thomas Eick, Sabine Hummert, Eckhard Schulz, Ralf Schmauder, Jana Schirmeyer, Thomas Zimmer, Klaus Benndorf

Scientific Reports 6; Article number: 2097410.1038/srep20974; published online: 02 09 2016; updated: 06 28 2018

The Acknowledgements section in this Article is incomplete.

“We would like to thank G. Ditze, G. Sammler, F. Horn, M. Händel, K. Schoknecht, S. Bernhardt, and A. Kolchmeier for technical assistance. This work was supported by grants of the Deutsche Forschungsgemeinschaft to K.B. and V.N. and of the Friedrich Schiller University to V.N.”

should read:

“We would like to thank G. Ditze, G. Sammler, F. Horn, M. Händel, K. Schoknecht, S. Bernhardt, and A. Kolchmeier for technical assistance. This work was supported by the Deutsche Forschungsgemeinschaft (TRR 166 ReceptorLight; project A5 to K.B; project B1 to V.N.) and by the Friedrich Schiller University to V.N.”

