# Supplementary material for: Quantifying the cooperative subunit action in a multimeric membrane receptor
Source: Sci Rep. 2016 Feb 9;6:20974. doi: 10.1038/srep20974 (PMC4746656; doi:10.1038/srep20974)
Supplement: Supplementary Information [file srep20974-s1.pdf]

Supplementary Information

to the manuscript

Quantifying the cooperative subunit action in a multimeric membrane receptor

by

Nisa Wongsamitkul[1], Vasilica Nache[1], Thomas Eick, Sabine Hummert, Eckhard Schulz, Ralf Schmauder, Jana Schirmeyer, Thomas Zimmer and Klaus Benndorf[*]

[*] E-Mail: Klaus.Benndorf@med.uni-jena.de

[1] These authors contributed equally to this work

Supplementary Figures

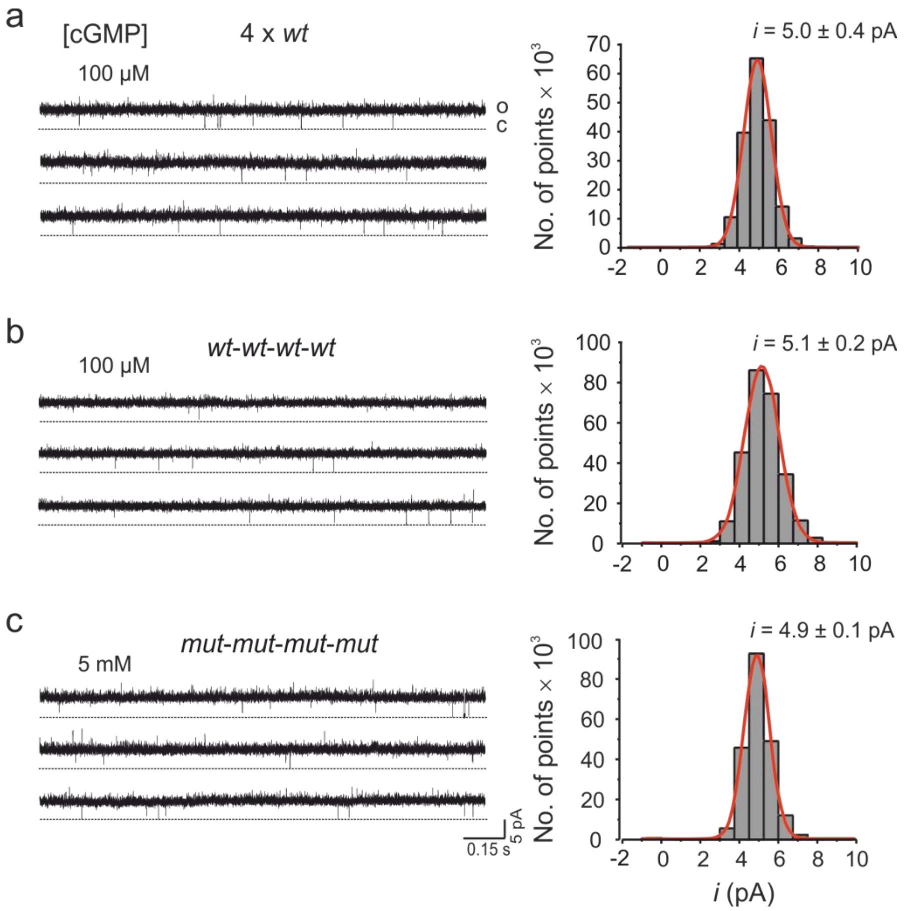

Supplementary Fig. 1. Single-channel recordings.

Three representative traces of single-channel activity and corresponding amplitude histograms for 4×*wt* (**a**), *wt-wt-wt-wt* (**b**) and *mut-mut-mut-mut* (**c**) at the indicated cGMP concentrations. "c" and "o" indicate the current level for closed and open channels, respectively. The amplitude of the single-channel current, *i* (see diagrams right) for all tested constructs is indistinguishable. Also the open probability for the constructs in **a** to **c** are closely similar (4×*wt*: 0.99±0.002, 100 µM cGMP; *N* = 4; *wt-wt-wt-wt*: 0.99±0.002, 100 µM cGMP, *N* = 4; *mut-mut-mut-mut*: 0.99±0.003, 5 mM cGMP, *N* = 5).

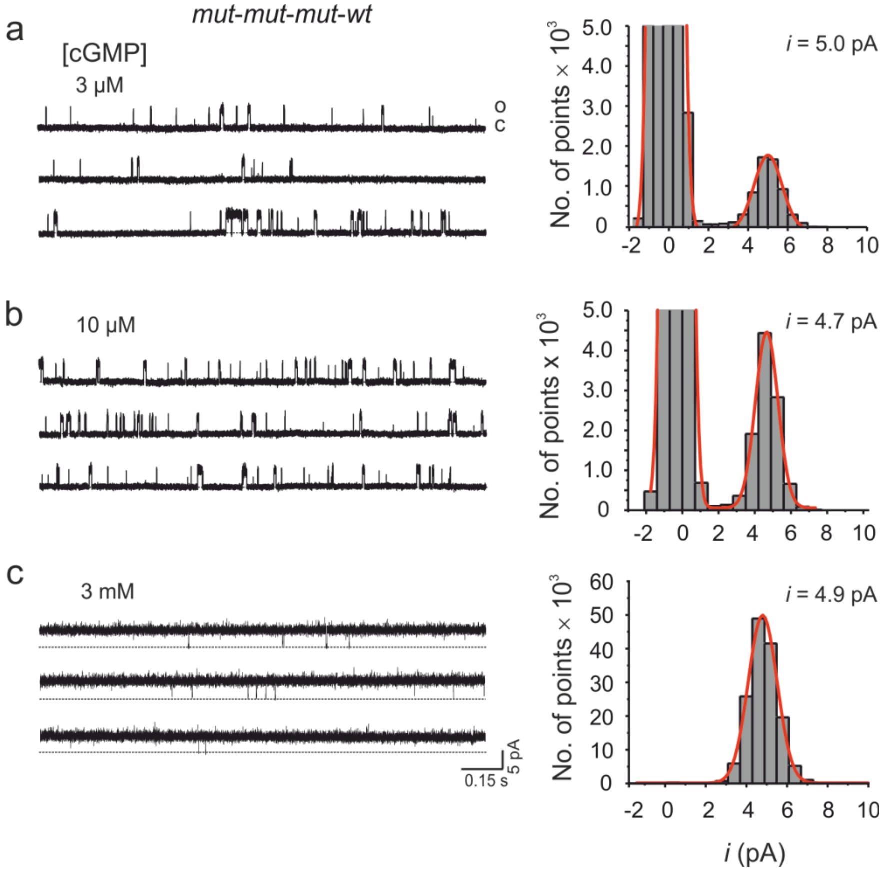

Supplementary Figure 2. Single-channel recordings of *mut-mut-mut-wt.*

Three representative traces of single-channel activity and corresponding amplitude histograms under control conditions and at the indicated three cGMP concentrations. "c" and "o" indicate the current level for closed and open channels, respectively. The amplitude of the single-channel current *i* does not depend on the cGMP concentration and is similar to 4 × *wt*-channels (Supplementary Fig. 1a).

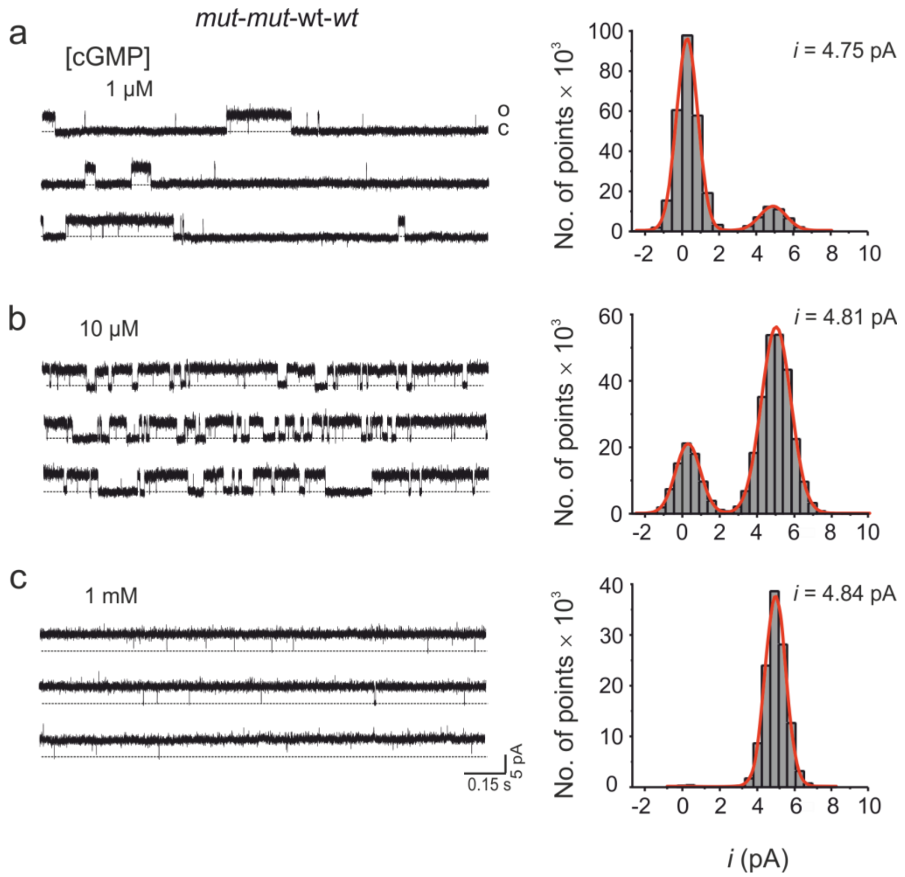

Supplementary Figure 3. Single-channel recordings of *mut-mut-wt-wt.*

Three representative traces of single-channel activity and corresponding amplitude histograms under control conditions and at the indicated three cGMP concentrations. “c” and “o” indicate the current level for closed and open channels, respectively. The amplitude of the single-channel current *i* does not depend on the cGMP concentration and is similar to *wt*-channels and all other concatamers (Supplementary Fig. 1a).

Supplementary Tables

Supplementary Table 1a. Effect of an increasing number of *mut*-subunits

Channel	EC_{50} / EC_{50H} (μM)	n / n_H	EC_{50L} (μM)	n_L	*A*	*N*
4×*wt*	1.92	2.04				5-9
4×*mut*	606.7	2.65				6-8
wt-wt-wt-wt	1.97	2.27				5-14
mut-wt-wt-wt	2.87	1.44				7-12
mut-mut-wt-wt	6.79	1.30				5-10
mut-mut-mut-wt	44.3	1 (fixed)	354.1	2.15	0.63	7-33
mut-mut-mut-mut	687.7	2.35				5-12

Fit parameters for concentration-activation relationships in Fig. 1a. The channels are either built of monomers (4×*wt*, 4×*mut*) or of concatenated subunits forming tetrameric concatamers (*wt-wt-wt-wt*, *mut-wt-wt-wt*, *mut-mut-wt-wt*, *mut-mut-mut-wt*, *mut-mut-mut-mut*). Apart from *mut-mut-mut-wt* the relationships were fitted with Equation 1. For *mut-mut-mut-wt* the relationship was fitted with Equation 2 yielding two components. *N* indicates the number of patches per data point.

Supplementary Table 1b. Concatamers assemble as tetrameric channels.

Channel	EC_{50} / EC_{50H} (μM)	n / n_H	EC_{50L} (μM)	n_L	*A*	*N*
wt-wt-wt-wt	1.97	2.27				5-14
mut-mut-mut-mut	687.7	2.35				5-12
wt-wt-wt-wt *plus* *mut-mut-mut-mut*	1.45	1.84	771.2	2.69	0.5	14-21

Fit parameters for concentration-activation relationships in Fig. 1b.

Supplementary Table 1c. The position of *wt*-subunits is irrelevant for the concatamer function.

**Channel**	**EC_{50} / EC_{50H} (µM)**	**n / n_H**	**EC_{50L} (µM)**	**n_L**	***A***	***N***
mut-mut-mut-wt	44.3	1 (fixed)	354.1	2.15	0.63	7-33
mut-mut-wt-mut	49.6	1 (fixed)	251.5	1.48	0.51	5-18
mut-wt-mut-mut	80.9	1 (fixed)	352.0	2.63	0.88	4-13
wt-mut-mut-mut	20.4	1 (fixed)	163.2	1.50	0.29	4-14
mut-mut-wt-wt	6.79	1.30				5-10
mut-wt-mut-wt	8.61	1.34				5-8
wt-mut-wt-mut	8.76	1.20				6-18
wt-wt-mut-mut	7.74	1.31				5-10

Fit parameters for concentration-activation relationships in Fig. 1c.

Supplementary Methods

Global fit of concentration-activation relationships

Concentration-activation relationships ($I/I_{\max} = f([\text{cGMP}])$) of the five concatamers *wt-wt-wt-wt*, *mut-wt-wt-wt*, *mut-mut-wt-wt*, *mut-mut-mut-wt*, and *mut-mut-mut-mut* were globally fitted with the five respective models shown in Figure 2a. The models are intimately coupled by common equilibrium constants. The open probabilities were computed according to the following equation:

$$P_{o,kwt}(L) = \frac{\mathbf{E} \bullet \mathbf{V}_{k\text{wt}}(L)}{(\mathbf{1}+\mathbf{E}) \bullet \mathbf{V}_{k\text{wt}}(L)}. \qquad \text{Equation S1}$$

A bold point indicates a dot product of two vectors, L represents the cGMP concentration in M, $\mathbf{E}$ is the vector of the equilibrium constants for the closed-open isomerizations E_0, E_1, E_2, E_3, and E_4 and $\mathbf{1}$ is a vector consisting of unities:

$$\mathbf{E} = \begin{pmatrix} E_0 \\ E_1 \\ E_2 \\ E_3 \\ E_4 \end{pmatrix}, \qquad \mathbf{1}+\mathbf{E} = \begin{pmatrix} 1+E_0 \\ 1+E_1 \\ 1+E_2 \\ 1+E_3 \\ 1+E_4 \end{pmatrix} \qquad \text{Equation S2}$$

For each $k = 4,\ldots,0$ a submodel specific vector $\mathbf{V}_{k\text{wt}}(L)$ is defined.

4*wt*-submodel:

$$\mathbf{V}_{4\text{wt}}(L) = \begin{pmatrix} 1 \\ 4LK_{\text{A1H}} \\ 6L^2K_{\text{A2H}}K_{\text{A1H}} \\ 4L^3K_{\text{A3H}}K_{\text{A2H}}K_{\text{A1H}} \\ L^4K_{\text{A4H}}K_{\text{A3H}}K_{\text{A2H}}K_{\text{A1H}} \end{pmatrix} \qquad \text{Equation S3}$$

3*wt*-submodel:

$$\mathbf{V}_{3\text{wt}}(L) = \begin{pmatrix} 1 \\ L(3K_{\text{A1H}} + K_{\text{A1L}}) \\ 3L^2K_{\text{A2H}}(K_{\text{A1H}} + K_{\text{A1L}}) \\ L^3K_{\text{A3H}}K_{\text{A2H}}(K_{\text{A1H}} + 3K_{\text{A1L}}) \\ L^4K_{\text{A4H}}K_{\text{A3H}}K_{\text{A2H}}K_{\text{A1L}} \end{pmatrix} \qquad \text{Equation S4}$$

2*wt*-submodel:

$$\mathbf{V}_{2\mathrm{wt}}(L)=\begin{pmatrix}1\\ 2L(K_{\mathrm{A1H}}+K_{\mathrm{A1L}})\\ L^2(K_{\mathrm{A2H}}K_{\mathrm{A1H}}+4K_{\mathrm{A2H}}K_{\mathrm{A1L}}+K_{\mathrm{A2L}}K_{\mathrm{A1L}})\\ 2L^3K_{\mathrm{A3L}}K_{\mathrm{A2H}}(K_{\mathrm{A1H}}+K_{\mathrm{A1L}})\\ L^4K_{\mathrm{A4L}}K_{\mathrm{A3L}}K_{\mathrm{A2H}}K_{\mathrm{A1H}}\end{pmatrix}$$ Equation S5

1*wt*-submodel:

$$\mathbf{V}_{1\mathrm{wt}}(L)=\begin{pmatrix}1\\ L(K_{\mathrm{A1H}}+3K_{\mathrm{A1L}})\\ 3L^2K_{\mathrm{A2L}}(K_{\mathrm{A1H}}+K_{\mathrm{A1L}})\\ L^3K_{\mathrm{A3L}}K_{\mathrm{A2L}}(3K_{\mathrm{A1H}}+K_{\mathrm{A1L}})\\ L^4K_{\mathrm{A4L}}K_{\mathrm{A3L}}K_{\mathrm{A2L}}K_{\mathrm{A1H}}\end{pmatrix}$$ Equation S6

0*wt*-submodel:

$$\mathbf{V}_{0\mathrm{wt}}(L)=\begin{pmatrix}1\\ 4LK_{\mathrm{A1L}}\\ 6L^2K_{\mathrm{A2L}}K_{\mathrm{A1L}}\\ 4L^3K_{\mathrm{A3L}}K_{\mathrm{A2L}}K_{\mathrm{A1L}}\\ L^4K_{\mathrm{A4L}}K_{\mathrm{A3L}}K_{\mathrm{A2L}}K_{\mathrm{A1L}}\end{pmatrix}$$ Equation S7

K_{A1H}, K_{A2H}, K_{A3H}, K_{A4H}, K_{A1L}, K_{A2L}, K_{A3L}, and K_{A4L} are the equilibrium association constants for the four high- and low affinity binding sites, respectively.

A Levenberg-Marquardt algorithm[1, 2] was used to optimize the parameters of the model. The χ^2 value was calculated from the fitted curves according to

$$\chi^2=\sum_{i=1}^{N}\frac{(P_{\mathrm{o,m}}(L_i)-P_{\mathrm{o,c}}(L_i))^2}{\sigma_i^2}.$$ Equation S8

$P_{\mathrm{o,m}}$ are the normalized measured open probabilities and $P_{\mathrm{o,c}}$ are the estimated open probabilities calculated according to Equation S1. The square of the deviations at a concentration L_i was weighted by the reciprocal squared values of the observed standard error

of mean (σ_i) before adding over all N data points of all concatamers. In the fit procedure χ^2 was minimized. The reduced chi-square, χ_r^2, was calculated by dividing χ^2 by the degrees of freedom (number of parameters subtracted from the number of data points). In the global fit χ_r^2 was 3.44.

The following strategy was used to reduce the number of parameters in the system of equations with 13 equilibrium constants:

1. The principle of microscopic reversibility delivered the following relation between the association constants

$$\frac{K_{\mathrm{A1H}}}{K_{\mathrm{A1L}}} = \frac{K_{\mathrm{A2H}}}{K_{\mathrm{A2L}}} = \frac{K_{\mathrm{A3H}}}{K_{\mathrm{A3L}}} = \frac{K_{\mathrm{A4H}}}{K_{\mathrm{A4L}}}. \qquad \text{Equation S9}$$

   Accordingly, three association constants could be expressed by other constants.

2. The equilibrium constants E_0 and E_5 were set according to the open probabilities determined from single-channel recordings being 1.7×10^{-5} and 9.9×10^{1}.
3. E_4 was set to E_5 to improve the determinateness.
4. To further increase the determinateness, K_{A3H} was set to K_{A4H}. According to Equation S9 K_{A3L} must then be equal to K_{A4L}. This assumption reduced the number of parameters by one.

As a result, only six equilibrium constants remained as free parameters.

Supplementary References

1. Brown KM, Dennis JE. Derivative-free analogues of the Levenberg-Marquardt and Gauss algorithmus or nonlinear least squares approximation. *Num Math* **18**, 289-297 (1972).

2. Press WH, Teukolsky SA, Vetterling WT, Flannery BP. Numerical Recipes in C: The Art of Scientific Computing. Cambridge University Press, 685 (2002).
